# Supplementary material for: Stretchable glove for accurate and robust hand pose reconstruction based on comprehensive motion data
Source: Nat Commun. 2024 Jul 11;15:5821. doi: 10.1038/s41467-024-50101-w (PMC11237015; doi:10.1038/s41467-024-50101-w)
Supplement: Supplementary file 1 — Supplementary Information [file 41467_2024_50101_MOESM1_ESM.pdf]

Supplementary Information

Stretchable Glove for Accurate and Robust Hand Pose Reconstruction Based on Comprehensive Motion Data

Myungsun Park[1,2†], Taejun Park[1,3†], Soah Park[4], Sohee John Yoon[1,3], Sumin Helen Koo[4]*, and Yong-Lae Park[1,3,5]*

†The authors equally contributed. *Corresponding authors (smkoo1@yonsei.ac.kr, ylpark@snu.ac.kr)

[1]Department of Mechanical Engineering, Seoul National University, Seoul 08826, Korea.

[2]Department of Mechanical and Aerospace Engineering, University of California San Diego, La Jolla, CA 92093, USA.

[3]Institute of Advanced Machines and Design, Seoul National University, Seoul, 08826, Korea

[4]Department of Clothing and Textiles, Yonsei University, Seoul 03722, Korea

[5]Institute of Engineering Research, Seoul National University, Seoul, 08826, Korea

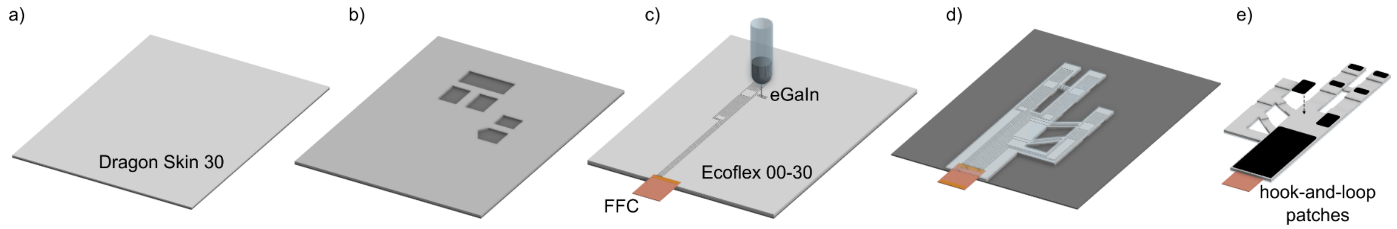

Supplementary Figure 1. Fabrication process of the soft sensing layer. (a) Aluminum plate coated with a layer of Dragon Skin 30. (b) Mold pattern cut out and removed using a laser cutter. (c) A layer of Ecoflex 00-30 coated on top of the mold, and eGaIn channels printed on the surface using an automatic dispensing system. (d) Outline of the sensor layer cut using the laser cutter. (e) Hook-and-loop patches bonded onto the anchoring points on the back side of the sensing layer.

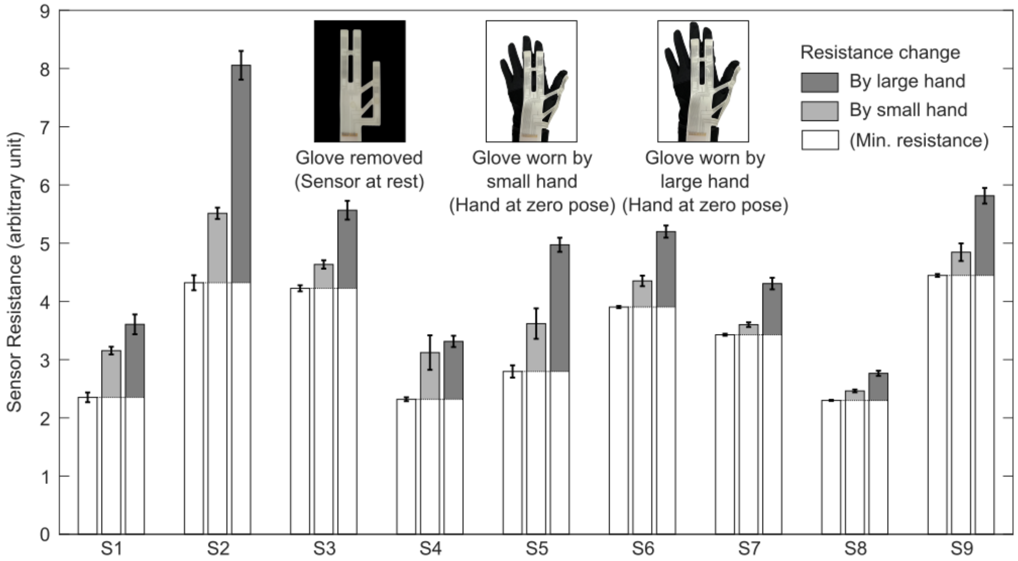

Supplementary Figure 2. Sensor signals for each of the nine sensors (S1-S9) measured at the initial unstretched state of the sensor (white, sample size=441), when worn on a small hand (light, sample size=337), and when worn on a large hand (dark, sample size=301), showing error bars (standard deviations) obtained over five trials. Sensor signals were measured with the hand at zero pose. Inset images depict the sensor in each of the three states. Source data are provided in the Source Data file.

Supplementary Table 1. Fabric property testing categories and fabrics selected for textile glove interface

No.	Test	Unit	Standard
1	Tensile strength	N	ISO 13934-2:2014 Grab Method<br>(3N, Mechanical Washing (30 ± 3) ℃, net drying)
2	Tensile elongation	%	ISO 13934-2:2014 Grab Method,<br>CRE Type tensile tester
3	Dimensional change in laundering	%	ISO 5077:2011 + ISO 6330:2021
4	Composition	%	ISO 1833-2:2020
5	Weight	g/㎡	ISO 3801:1977 Method 5
6	Thermal transmittance	㎡·K/W	ASTM D 1518-2014 Option 1 : Still Air Condition
7	Bursting strength	kPa	ISO 13938-1:2019<br>Diaphragm Method
8	Abrasion strength	RUBS	ISO 12947-2:2016 Martindale Method
9	Elongation recovery rate	%	ASTM D 2594-2004(2016) / ASTM D3107-07(2019)<br>Sample 1<br>1. Gage length: 250 mm<br>2. Applied load: 1.8 kg (4.0 lbf)<br>Sample 2,3<br>1. Gage length: 125 mm<br>2. Applied load: 44.5 N (10 lbf)<br>3. Elongation distance:<br>Wale 35%, Course 60% (Form-fitting)
10	Pilling	grade	ISO 12945-1:2020 ICI Pilling Box Method
11	Air permeability	mm/s	ISO 9237:1995

Sample No.	Fabric	Composition	Color
1	Woven Fabric	PE=100	Black
2	Air permeable fabric	NY/SP=87.9/12.1	Black
3	Elastic Fabric	NY/SP=89.2/10.8	Black

Supplementary Table 2. Fabric property test results

Sample No.	Tensile strength (N)	Tensile elongation (%)	Dimensional change in laundering (%)	Composition (%)			Weight (g/m²)	Thermal transmittance (m² K/W)	Bursting strength (kPa)	Abrasion strength (RUBS)
				PE	NY	SP				
1	WP 780 WF 570	WP 8.9 WF 8.2	WP-0.9 WF-0.5	100	-	-	166	0.016	1570	500 ↑ (Final point: when the two strands of thread are completely cut off)
2	WP 270 WF 310	WP 98.7 WF 56.4	W-1.1 C-4.8	-	87.9	12.1	205	0.010	Does not burst	500 ↑ (Final point: when a hole occurs in the fabric)
3	WP 380 WF 440	WP 77.6 WF 78.9	W-2.4 C-1.1	-	89.2	10.8	235	0.008	Does not burst	500 ↑ (Final point: when a hole occurs in the fabric)

	Elongation recovery rate (%)					Pilling (grade)	Air permeability (mm/s)
	Elongation	Residual elongation (after 30 sec)	Residual elongation (after 60 min)	Elongation Recovery (after 30 sec)	Elongation Recovery (after 60 min)		
1	WP 1.2 WF 1.4	WP 0.3 WF 0.3	WP 0.2 WF 0.2	WP 79 WF 79	WP 85 WF 85	4-5	0.550
2	W 88 C 44	W 4.8 C 14	W 3.5 C 9.9	W 86 C 77	W 90 C 84	4-5	295
3	W 65 C 47	W 7.2 C 20	W 3.5 C 14	W 79 C 67	W 90 C 77	4-5	1380

Supplementary Note 1. Bone lengths measurement for different hand size

The ground truth lengths of the finger bones were measured for each subject using the commercial image analysis software ProAnalyst (Xcitex). Subjects were instructed to place their hands flat on the test table and maintain the zero-pose, and the hand was photographed from the top. To reduce distortions due to inconsistent point of view, the camera was positioned horizontally using a digital level. Positions of each finger joint were marked by identifying the characteristic features of joint creases and protruding contours of the joint bones, and the distance between successive markers were calculated as each bone length.

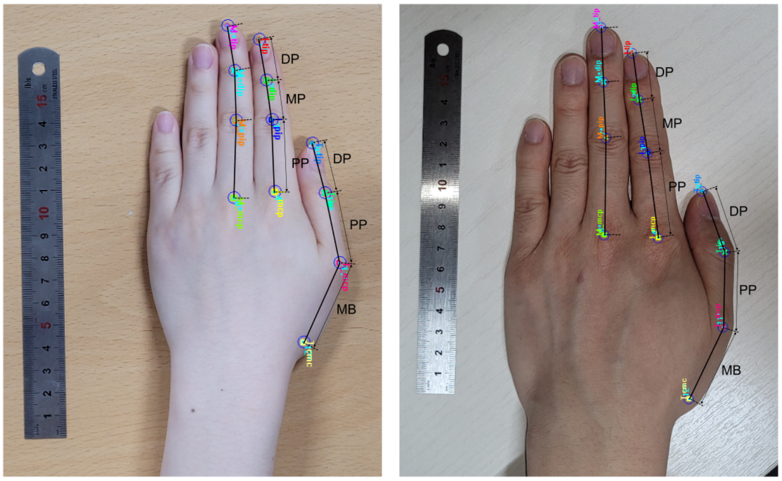

Supplementary Figure 3. Finger bone length measurement method using ProAnalyst, indicating position of each joints marked

Supplementary Table 3. Initial bone lengths estimation results (Mean absolute errors) of 13 subjects and all bones. Source data are provided in the Source Data file.

Subject No.	Mean absolute error (mm)									
	I DP	I MP	I PP	M DP	M MP	M PP	T DP	T PP	T MB	Mean
1	2.75	0.09	3.63	3.48	1.17	4.96	1.99	0.55	0.81	2.16
2	2.55	1.40	1.74	2.66	1.24	7.37	1.46	2.13	0.31	2.32
3	3.66	3.88	5.00	3.35	3.02	8.19	4.55	1.06	0.07	3.64
4	2.56	1.51	5.46	2.69	0.57	2.27	1.86	3.27	1.65	2.43
5	0.52	3.25	1.24	2.46	0.79	0.28	2.38	3.07	3.66	1.96
6	2.53	0.97	5.47	0.67	0.69	0.46	1.22	7.87	1.08	2.33
7	2.12	0.84	0.59	3.63	0.87	1.78	1.20	3.39	4.22	2.07
8	0.20	1.35	0.30	1.96	0.78	0.77	0.18	0.11	0.34	0.66
9	0.13	0.05	2.93	0.85	1.36	0.35	1.68	1.42	3.40	1.35
10	0.28	3.42	2.53	0.73	4.77	3.29	2.38	2.42	0.88	2.30
11	2.23	1.73	2.89	1.43	3.11	2.64	1.28	0.56	0.49	1.82
12	0.43	0.89	3.96	0.89	1.86	3.25	0.03	1.37	3.68	1.82
13	2.16	2.42	0.50	3.21	0.81	0.38	2.50	1.10	4.45	1.95
Mean	1.70	1.68	2.79	2.15	1.62	2.77	1.75	2.18	1.93	2.06
St. Dev.	1.21	1.23	1.85	1.13	1.26	2.65	1.14	2.02	1.68	1.57

Supplementary Table 4. Refined bone lengths estimation results of 5 subjects and all bones. “True”, “Initial”, and “Refined” represent the true bone lengths, initial estimation errors (RMSE), and refined estimation errors (RMSE), respectively.

Subject No.	Bone lengths (mm)														
	I DP			I MP			I PP			M DP			M MP		
	True	Initial	Refined	True	Initial	Refined	True	Initial	Refined	True	Initial	Refined	True	Initial	Refined
1	18.43	3.66	3.10	17.73	3.87	2.34	32.34	5.00	4.17	20.16	3.35	2.05	22.25	3.02	3.01
2	22.79	2.55	3.31	22.43	1.40	0.83	36.59	1.74	2.13	24.93	2.66	4.13	27.17	1.24	0.91
3	21.14	2.15	0.22	23.49	2.42	0.14	41.44	0.50	0.21	22.82	3.21	1.04	27.83	0.81	0.05
4	23.46	2.23	1.79	20.05	1.73	2.00	39.80	2.89	2.67	24.21	1.43	0.94	23.41	3.11	2.52
5	20.50	0.43	0.05	21.00	0.90	0.84	33.04	3.96	3.40	21.31	0.89	0.17	25.80	1.86	1.23
Subject No.	M PP			T DP			T PP			T MB			Mean		
	True	Initial	Refined	True	Initial	Refined	True	Initial	Refined	True	Initial	Refined	True	Initial	Refined
1	34.71	8.19	7.70	22.73	4.56	5.02	32.04	1.06	1.11	38.68	0.05	0.02	31.44	2.32	2.29
2	47.91	7.36	5.28	28.34	1.47	1.66	33.46	2.13	2.09	39.35	0.31	0.31	26.56	3.64	3.17
3	48.00	0.38	1.02	28.83	2.49	0.13	38.85	1.10	1.17	42.61	4.45	4.36	30.83	1.82	1.52
4	44.60	2.64	2.03	28.10	1.28	0.71	33.53	0.56	0.57	40.32	0.49	0.45	27.91	1.82	1.67
5	37.81	3.25	2.97	25.49	0.04	1.36	31.19	1.37	1.37	35.09	3.68	3.62	32.78	1.95	0.93

Supplementary Table 5. Comparison of hand tracking technologies. Joint angle errors are in degrees and fingertip position errors are in millimeters.

	Sensors	Reported accuracy (error)	Kinematic parameter measurements	(Per-user) Calibration-free	Number of fingers	Speed (Hz)	User Interface	Other Features
Pro Fidelity Glove, StretchSense [1]	Strain	N/A			5	120	✓	• $6,995<br>• Machine-washable
Glauser et al. [2]	Strain	5.30—8.80°		✓	5	60		• 43 sensors
Kim et al. [3]	Strain	N/A		✓	1	30		• Direct printing<br>• Non-reusable
Lee et al. [4]	Vision + IMU	10.69—12.68 mm	✓	✓	5	100		• Head mounted stereo camera
Leap Motion Controller, Ultraleap [5]	Vision	7.9—16.5°	✓	✓	5	115		• $139
MediaPipe [6,7]	Vision	9.98° (upper limbs)	✓	✓	5	60	✓	• Open Source
Smartglove, Rokoko [8,9]	Magnetic + IMU	N/A			5	100	✓	• $1,495
Quantum Mocap Metaglove, Manus [10,11]	Magnetic	< 1 mm	✓	✓	5	120	✓	• $5,384<br>• Machine-washable
This work	Strain	3.91—7.14°, 3.24—4 02 mm	✓	✓	3	60		

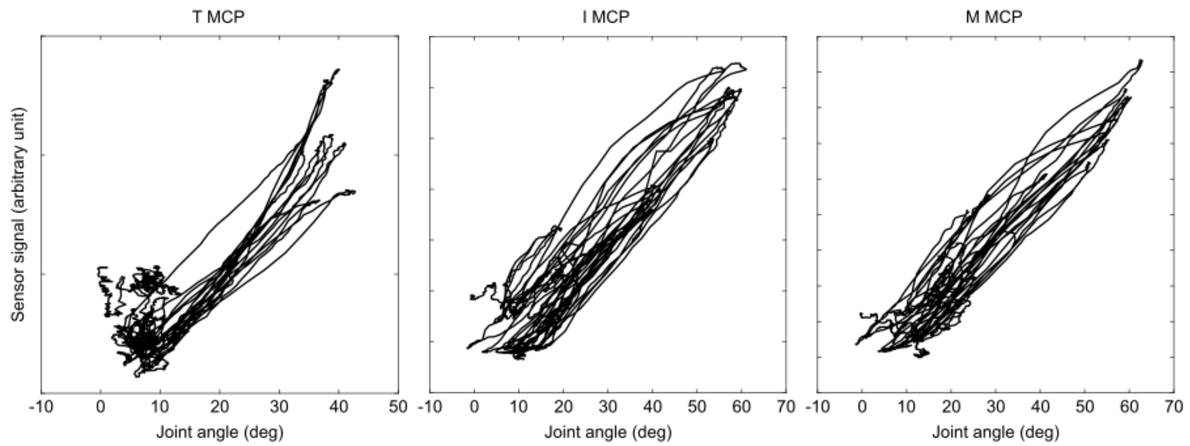

Supplementary Figure 4. Sensor signal generated by corresponding (Figure 1c) flexion angles of thumb MCP, index and middle finger MCP joints. Source data are provided in the Source Data file.

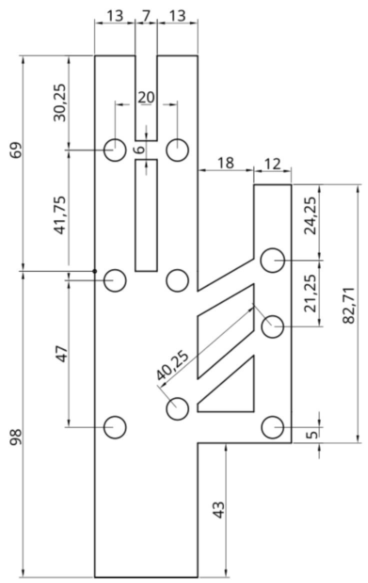

Supplementary Figure 5. Dimensions of the soft sensing layer, values given in mm. Anchor points (position of hook-and-look patches) are represented as circles.

Supplementary Video 1. Free hand motions wearing the sensing glove

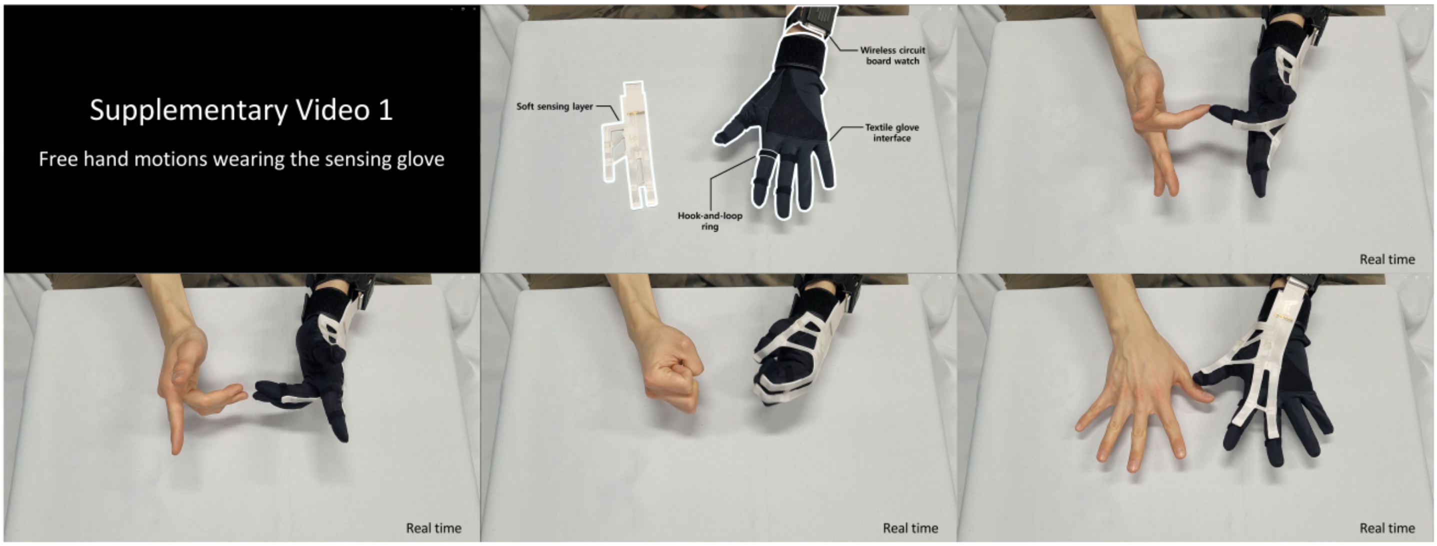

Supplementary Video 2. Hand pose reconstruction for free hand motion and grasping

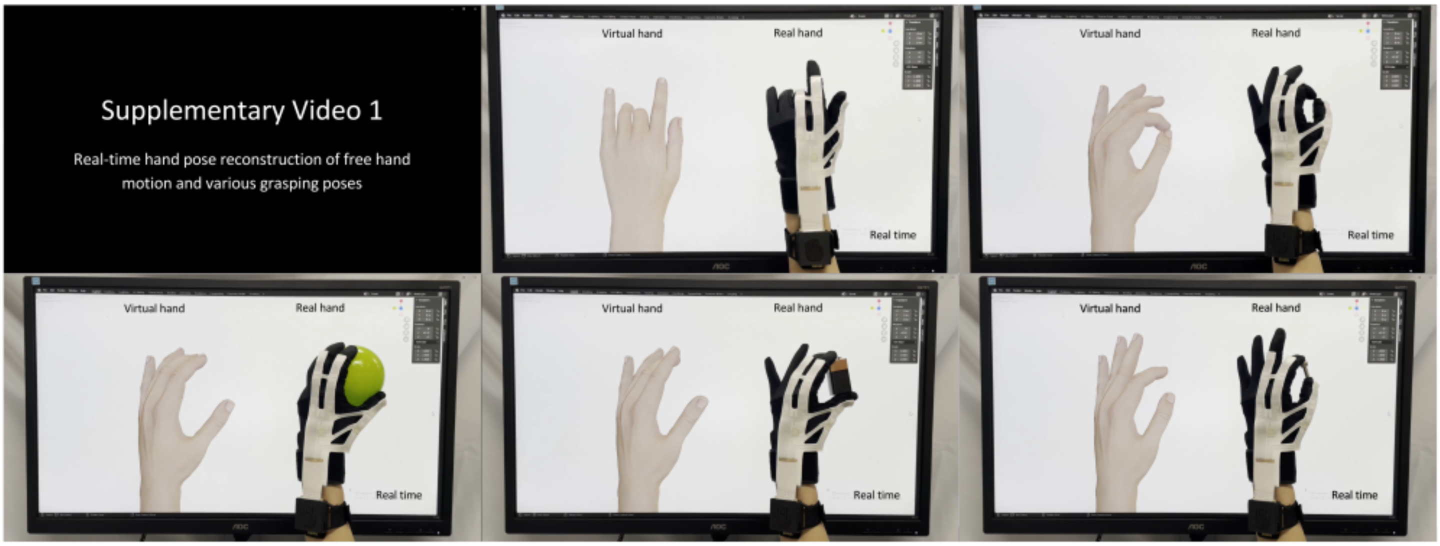

Supplementary Video 3. Modified Kapandji test

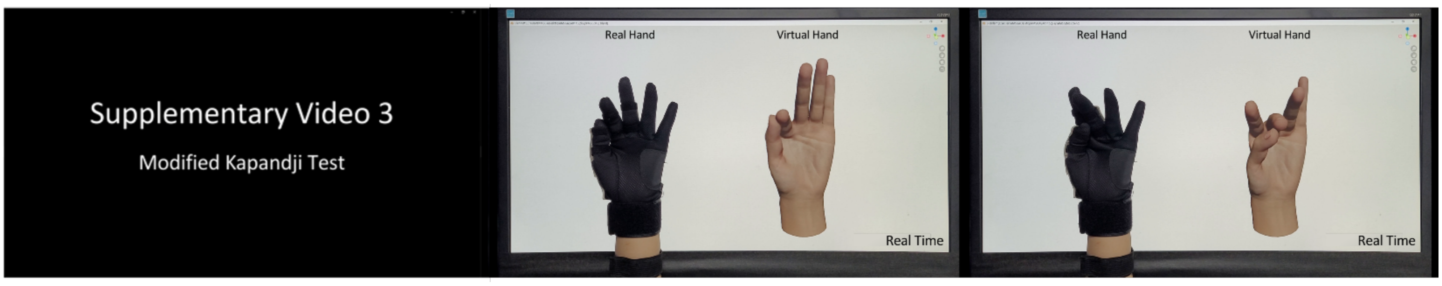

Supplementary Video 4. Application 1: Typing of virtual number pad

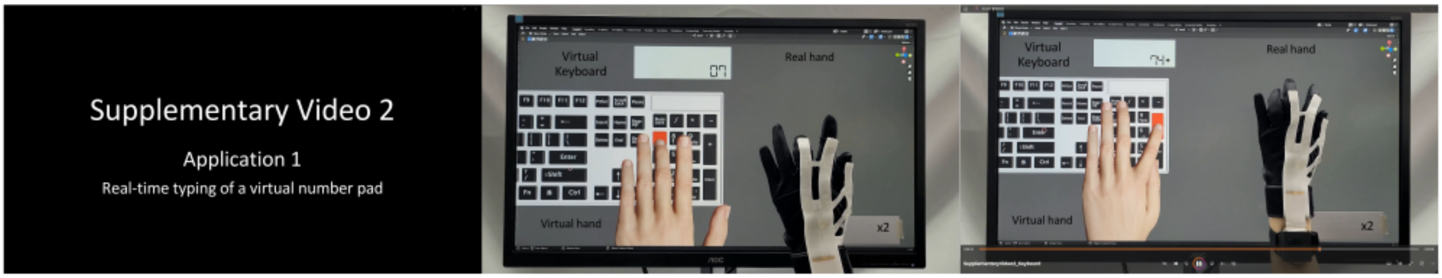

Supplementary Video 5. Application 2: Virtual shadowgraphy

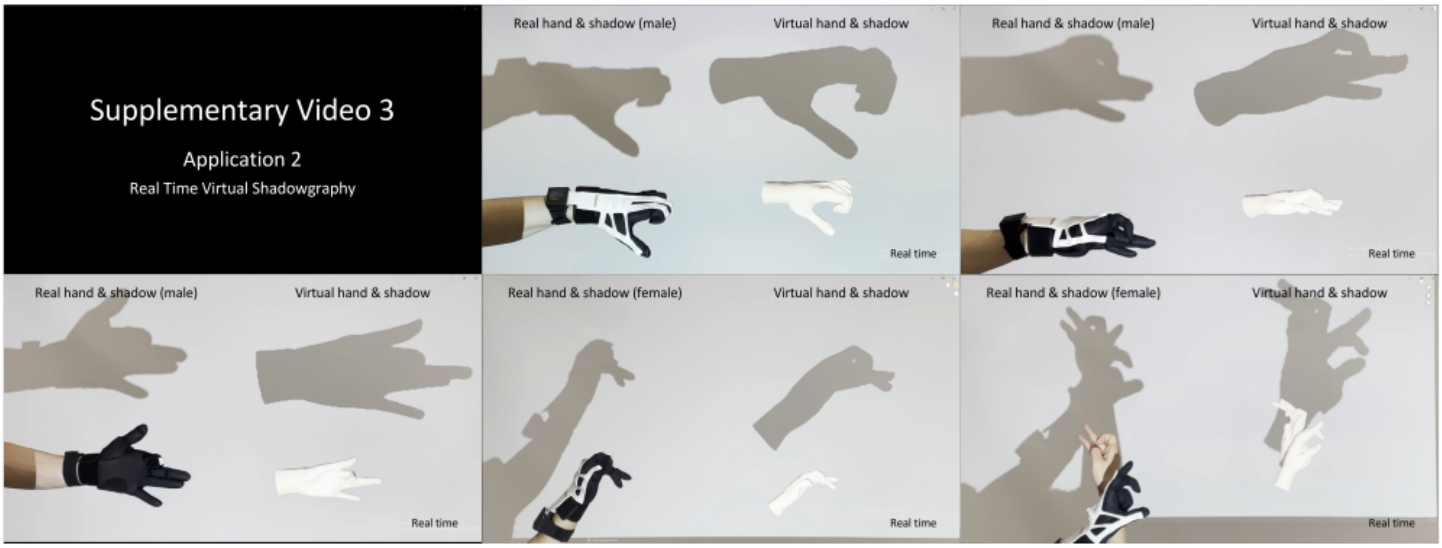

Supplementary Video 6. Application 3: Real-time teleoperation of a dexterous robotic hand

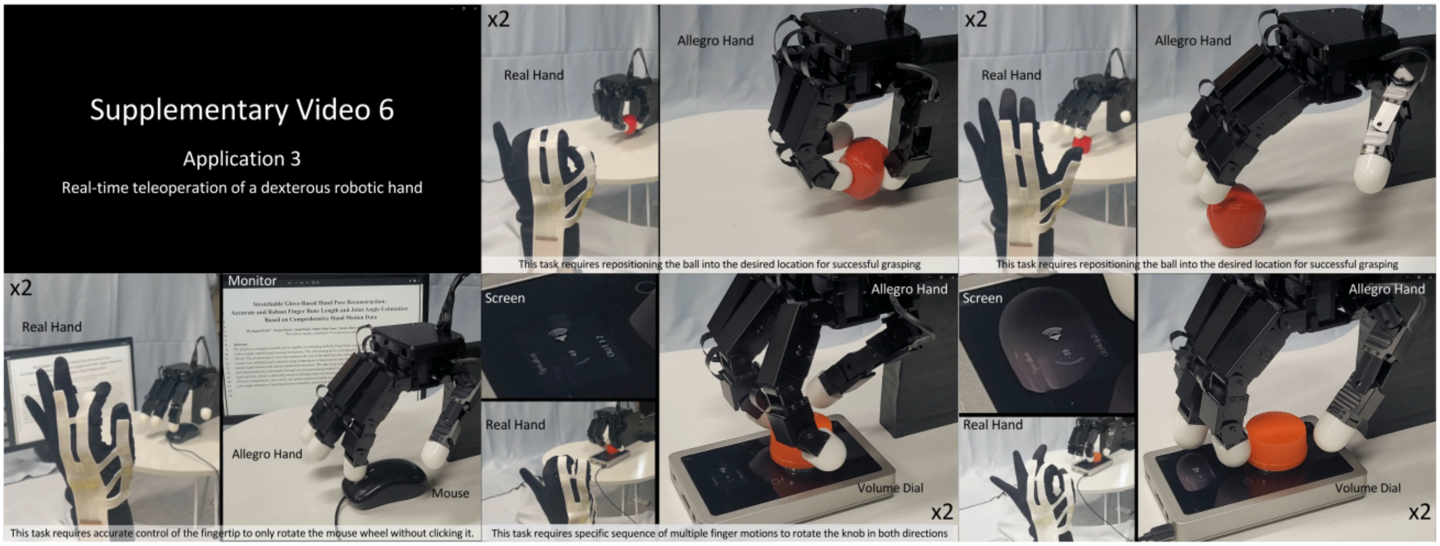

References

1. StretchSense, MoCap Pro Fidelity Glove, https://stretchsense.com/mocap-pro-fidelity-glove-2/ (2024).
2. Glauser, O., Wu, S., Panozzo, D., Hilliges, O. & Sorkine-Hornung, O. Interactive hand pose estimation using a stretch-sensing soft glove. *ACM Transactions on Graphics (ToG)*, **38(4)**, 1-15 (2019).
3. Kim, K. et al. A substrate-less nanomesh receptor with meta-learning for rapid hand task recognition. *Nature Electronics*, **6(1)**, 64-75 (2023).
4. Lee, Y., Do, W., Yoon, H., Heo, J., Lee, W., & Lee, D. Visual-inertial hand motion tracking with robustness against occlusion, interference, and contact. *Science Robotics*, **6(58)**, eabe1315 (2021).
5. Mizera, C., Delrieu, T., Weistroffer, V., Andriot, C., Decatoire, A., & Gazeau, J. P. Evaluation of hand-tracking systems in teleoperation and virtual dexterous manipulation. *IEEE Sensors Journal*, **20(3)**, 1642-1655 (2019).
6. Lafayette, T. B. D. G. et al. Validation of angle estimation based on body tracking data from RGB-D and RGB cameras for biomechanical assessment. *Sensors*, **23(1)**, 3 (2023).
7. Mediapipe, Mediapipe Solutions Guide, https://developers.google.com/mediapipe/solutions/guide.
8. Rokoko, Smartgloves, https://www.rokoko.com/products/smartgloves (2024).
9. Ji, B. et al. Flexible Strain Sensor-Based Data Glove for Gesture Interaction in the Metaverse: A Review. *International Journal of Human–Computer Interaction*, 1-20 (2023).
10. Manus, Quantum Mocap Metagloves, https://www.manus-meta.com/products/quantum-mocap-metagloves (2024).
11. Achenbach, P., Laux, S., Purdack, D., Müller, P. N., & Göbel, S. Give Me a Sign: Using Data Gloves for Static Hand-Shape Recognition. Sensors, **23(24)**, 9847 (2023).
