# Supplementary material for: Stretchable glove for accurate and robust hand pose reconstruction based on comprehensive motion data
Source: Nat Commun. 2024 Jul 11;15:5821. doi: 10.1038/s41467-024-50101-w (PMC11237015; doi:10.1038/s41467-024-50101-w)
Supplement: Supplementary file 3 — Description of Additional Supplementary Files [file 41467_2024_50101_MOESM3_ESM.pdf]

Description of Additional Supplementary Files

Supplementary Video 1. Free hand motions wearing the sensing glove.

Supplementary Video 2. Real-time hand pose reconstruction of free hand motion and various grasping poses. 1) Free hand motions. 2) Sphere grasp 3) Tripod grasp. 4) Precision grip.

Supplementary Video 3. Modified Kapandji test. Real-time reconstruction of the thumb reaching various bones of the index and middle fingers.

Supplementary Video 4. Application 1 Real-time typing of a virtual number pad. 1) Typing a five digit number. 2) Simple arithmetic calculation.

Supplementary Video 5. Real-time virtual shadowgraphy.

Supplementary Video 6. Real-time teleoperation of a dexterous robotic hand. 1) Relocating and grasping a ball. 2) Scrolling a mouse wheel. 3) Adjusting a volume dial (reducing and increasing the volume)
